# Multidimensional Differences Between Athletes of Endurance, Strength, and Intermittent Sports: Body Composition, Diet, Resting Metabolic Rate, Physical Activity, Sleep Quality, and Subjective Well-Being

**DOI:** 10.3390/nu17071172

**Published:** 2025-03-28

**Authors:** Marcos Rueda-Cordoba, Juan J. Martin-Olmedo, Sergio Espinar, Jonatan R. Ruiz, Lucas Jurado-Fasoli

**Affiliations:** 1 Department of Physical Education and Sports, Faculty of Sports Science, Sport and Health University Research Institute (iMUDS), University of Granada, 18071 Granada, Spain; mruedacordoba@gmail.com (M.R.-C.); martinolmedo@ugr.es (J.J.M.-O.); ruizj@ugr.es (J.R.R.); 2 Department of Health Research, Icen Canary University, 38002 Santa Cruz de Tenerife, Spain; sergio@sergioespinar.com; 3 Department of Physiology, Faculty of Medicine, University of Granada, 18001 Granada, Spain; 4 Faculty of Health Sciences, Catholic University of Murcia, 30107 Murcia, Spain; 5 Instituto de Investigación Biosanitaria (Ibs), 18012 Granada, Spain; 6 Centro de Investigación Biomédica en Red Fisiopatología de la Obesidad y Nutrición (CIBERobn), Instituto de Salud Carlos III, 28029 Madrid, Spain

**Keywords:** nutrition, energy, macronutrients, recovery, conditioning, fat-free mass, fat mass, energy expenditure

## Abstract

**Background/Objectives:** Sports performance is influenced by a complex interplay of physical, physiological, and psychological factors, which differ across disciplines. Thus, this study aims to identify and compare the distinct multidimensional profiles (i.e., body composition, diet, resting metabolic rate, physical activity, sleep quality, and subjective well-being) of athletes from different sports disciplines (i.e., endurance, strength, and intermittent sports). **Methods**: This study included 77 athletes (28 women) classified as endurance (n = 40), strength (n = 12), or intermittent (n = 25). Body composition was assessed by dual-energy X-ray absorptiometry, bioimpedance, and anthropometry, diet was determined using dietary recalls, resting metabolic rate was assessed by indirect calorimetry, physical activity and sleep quality were determined by a wrist-worn accelerometer, and subjective well-being was determined through validated questionnaires. **Results**: Strength and intermittent athletes had greater body weight, lean mass, and bone parameters than endurance athletes, whereas intermittent athletes showed higher adiposity than endurance levels (all *p* ≤ 0.008). The endurance group did not exhibit a higher intake of fats or proteins compared to the strength and intermittent groups; however, it did demonstrate a higher consumption of carbohydrates (*p* ≤ 0.016). No differences were observed in resting metabolic rate, sleep quality, and subjective well-being, though endurance athletes demonstrated higher levels of vigorous physical activity than strength athletes (*p* = 0.014). **Conclusions**: In conclusion, we reveal a distinct physiological phenotype between strength, intermittent, and endurance athletes in terms of body composition, dietary intake, and physical activity levels. These findings underscore the necessity for tailored training and nutrition protocols depending on the sports discipline.

## 1. Introduction

Sports performance is multifactorial and complex, relying on the interaction of numerous physical (e.g., strength, endurance, coordination), physiological (e.g., body composition, energy systems, recovery, adaptation), or psychological (e.g., motivation, focus, mood) factors [1]. Understanding these factors holistically and optimizing them through tailored training, recovery, and mental conditioning is essential for maximizing sports performance [2].

Body composition is a key determinant of athletic performance, with lower fat and higher muscle mass associated with improvements in strength, power, and endurance [3]. Additionally, nutrition plays a crucial role, as adequate macronutrient intake supports these physical attributes and enhances training capacity, recovery, and injury prevention [4]. Furthermore, sleep is indispensable for optimizing performance, affecting not only physical recovery but also mental health and overall well-being [5]. Monitoring these interconnected variables is vital for maximizing athletic success and safeguarding long-term health.

However, each sports discipline has specific characteristics and physiological demands [2]. Endurance athletes are characterized by higher cardiovascular and pulmonary capacity, increased maximal oxygen consumption, and sustained muscular adaptations such as greater capillary density and improved metabolic efficiency [6]. Strength athletes are characterized by increased muscle strength and size through enhanced muscle protein synthesis, improved neuromuscular coordination, stronger bones and connective tissues, and metabolic adaptations [6]. Intermittent sports, such as football, handball, or basketball, combine aerobic and anaerobic systems, enhancing cardiovascular endurance, agility, speed, and muscular strength [7]. These sports promote adaptations such as improved maximal oxygen consumption, faster recovery from high-intensity efforts, better coordination, and increased muscle power, while also fostering cognitive skills like decision-making and teamwork [7]. Consequently, the physiological and psychological profiles of athletes can vary considerably depending on the demands of their specific sport.

Indeed, strength athletes typically possess greater muscle mass compared to athletes from other disciplines, such as endurance or intermittent sports athletes [8]. Additionally, strength athletes tend to expend more calories at rest, due to their higher body weight and greater fat-free mass (FFM) [8]. Conversely, endurance athletes are often characterized by lower bone mineral density (BMD) and fat mass (FM) levels [9]. Regarding the nutritional habits of different athletes, endurance athletes report a higher intake of carbohydrates compared to other groups; however, strength athletes show the highest intake of protein [10]. Beyond these distinctions, there is a notable scarcity of studies that comprehensively compare physiological and psychological differences between various sports disciplines. Understanding phenotypic differences across sports disciplines is crucial for optimizing athlete performance and health, as each discipline demands specific physiological and psychological adaptations, and would help to develop tailored training and nutrition protocols.

Therefore, this study aims to identify and compare the distinct physiological and psychological profiles of athletes from different sports disciplines (i.e., endurance, strength, and intermittent sports). Here we will focus on body composition, diet, resting metabolic rate (RMR), handgrip strength, physical activity, sleep quality, and subjective evaluations of health and wellness, including perceptions of recovery, eating disorder symptoms, overtraining, stress, and health-related symptoms.

## 2. Materials and Methods

### 2.1. Participants and Study Design

A total of 77 athletes, comprising 49 men and 28 women, were included in this cross-sectional study. The participants were recruited through social media and the dissemination of the study within various sports institutions of Granada. These athletes represented various sports disciplines and actively competed at regional, national, or international levels. Each participant attended the Sport and Health University Research Center (iMUDS) on two separate occasions. During the first visit, a detailed briefing was conducted in which the study protocol was thoroughly explained, and informed consent was obtained. Following this, a comprehensive assessment was performed, which included measurements of body composition, muscular strength, resting metabolic rate, and a 24 h dietary recall.

The study protocol received approval from the Human Research Ethics Committee of the “Junta de Andalucía” (No. 0909-N-22). All participants were fully informed about the procedures, and potential risks, and provided written consent before participation. The inclusion criteria were as follows: (1) participants were required to be between 18 and 35 years of age, (2) actively competing and federated in any sports discipline for a minimum of two years, (3) female participants were required not to be pregnant or breastfeeding, (4) all participants had no history of cardiovascular events or chronic diseases, and (5) participants had no history of tobacco or alcohol abuse or illicit use of drugs, medications that alter the results of the study, or history of alcohol abuse.

Participants were classified in endurance (n = 40; swimming, cycling, running, triathlon, trail running), strength (n = 12; powerlifting, bodybuilding, weightlifting, crosstraining), or intermittent athletes (n = 25; football, handball, basketball, hockey, volley, combat sports) depending on the competitive sports discipline.

Participants were instructed to abstain from strenuous physical activity for 24 h before the assessments and to present themselves in a fasting state, having refrained from alcohol and stimulant consumption, to ensure measurement accuracy. Upon completing the initial assessment battery, participants were provided with wrist triaxial accelerometers, which they were instructed to wear continuously for nine days. After this period, participants returned to the laboratory to return the accelerometers and record their physical activity and sleep quality.

### 2.2. Physiological and Psychological Assessments

#### 2.2.1. Body Composition and Anthropometrics

Body weight was measured to the nearest 0.1 kg and height to the nearest 0.1 cm, using a model 799 scale and a stadiometer, respectively (Seca model 799, Electronic Column Scale, Hamburg, Germany), without shoes and with light clothing. Body mass index was calculated from weight and height (kg/m^2^).

Body composition was assessed using three distinct methods: Dual-Energy X-ray Absorptiometry (DXA), bioelectrical impedance analysis (BIA), and anthropometry. These methods provided comprehensive measurements of body fat, lean mass, and other key body components. Lean mass, lean mass index (LMI), fat-free mass (FFM), fat mass (FM), fat mass index (FMI), android FM, gynoid FM, visceral adipose tissue (VAT) mass, total body bone mineral density (BMD), total body bone mineral content (BMC), and total body BMD Z-score were measured by DXA using a Discovery Wi device (Hologic, Bedford, MA, USA) equipped with analysis software (APEX version 4.0.2). Lean and fat mass indices were expressed as kg/m^2^; fat mass was also expressed as a percentage of body weight. During the scan, participants lay motionless on a table while a scanning arm moved over the body, capturing detailed cross-sectional images. The DXA scan was performed under standardized conditions, with strict adherence to the manufacturer’s guidelines for calibration and quality control (test-retest ICC ≈ 0.98–0.99 for total body composition [11,12]).

Bioelectrical measurements of resistance and reactance were taken at 50 kHz using a Tanita (MC-980 MA N-PLUS, Tokyo, Japan). Electrodes were strategically placed on the participants, hands, and feet, and a small safe electrical current was passed through the body. The resistance to this current, combined with factors such as height, weight, age, and sex, was used to estimate body composition. The following outcomes were obtained from the procedure: FFM, FM, muscle mass, and phase angle (within-day CV < 1% for total body composition [13]).

Kinanthropometric measurements were taken according to the ISO 7250-1:2017 [14] and the International Society for the Advancement of Kinanthropometry (ISAK) standard [15]. The measurements taken were as follows: four basic measurements (body mass, height, sitting height), eight skinfolds (biceps, triceps, subscapular, iliac crest, supraspinal, abdominal, thigh, and calf), ten circumferences (head, forearm, arm relaxed, arm flexed and contracted, chest, waist, hips, thigh middle, and calf) and six breadths (biacromial, biiliocristal, antero-posterior chest depth, transverse chest, humerus, and femur). At the same time, the sum of skinfolds six and eight was used, and adipose tissue was calculated through Kerr’s equation [16]. The anthropometric material used to measure circumference was a LUFKIN inextensible metal tape (APEX TOOL GROUP, Sparks, MD, USA); an anthropometer to measure large breadths and segmometer (CESCORF, Porto Alegre, Brazil), a small anthropometer, and a skinfolds caliper of 0.2 mm precision (HOLTAIN Ltd., Crymych, UK). All kinanthropometric measurements were measured two or three times by an anthropometrist level 2 and 3 accredited by ISAK (MR-C and JJM-O), depending on whether the technical error of measurement (TEM) between the first two measurements was greater than 5% in skinfolds and 1% for the rest of the measurements, taking the mean or median, respectively, for subsequent analysis. The somatotype was assessed using the Heath–Carter method, which calculates the three individual components of somatotype (endomorphy, mesomorphy, and ectomorphy).

#### 2.2.2. Dietary Assessment

Habitual food intake was assessed through three non-consecutive 24 h dietary recalls (two weekdays and one weekend day/holiday), conducted via face-to-face interviews by a trained and qualified researcher dietician. The interviews followed a meal-sequence approach, involving a detailed assessment and description of all foods and beverages consumed. To enhance the accuracy of portion size estimation, photographs depicting various portion sizes were utilized during the interviews. Energy and nutrient intakes were subsequently obtained using the EvalFINUT^®^ software 2.0., which draws upon data from both the United States Department of Agriculture (USDA) and the Spanish Food Composition Database (BEDCA). Energy availability (EA) was calculated as energy intake minus exercise energy expenditure divided by FFM.

#### 2.2.3. Resting Metabolic Rate

Resting metabolic rate was estimated through indirect calorimetry. In short, participants lay motionless in a supine position on a bed, covered with a bed sheet for at least 20 min to acclimate before the RMR measurement. Then, gas exchange at rest was collected and measured by using a ventilated canopy hood attached to the Quark CPET RMR (COSMED, Rome, Italy) metabolic cart for 30 min. Gas exchange data were downloaded at a sample frequency of 5 s, and VO_2_ and volume of carbon dioxide production (VCO_2_) were used to estimate energy expenditure. For exchange data, the first 5 min of measurements was discarded [17] and the average VO_2_ and VCO_2_ were calculated (CV for VO_2_ ≈ 5.5% and CV for VCO_2_ ≈ 8.7% [18]). EE was estimated using Weir’s abbreviated equation considering zero nitrogen excretion [19].

#### 2.2.4. Handgrip Strength

The handgrip strength was assessed using a portable hand dynamometer Takei 5401 digital Grip-D hand dynamometer (Takei, Tokyo, Japan). Participants were instructed to stand with the shoulder of the testing side slightly abducted, allowing the corresponding arm to hang freely (~10° from the body) without elbow flexion. They were then prompted to gradually and continuously exert maximal grip strength. The dynamometer automatically recorded the peak force. Each participant completed the test twice, alternating between hands, with a 1 min rest period between attempts. The grip span was consistent for male participants but adjusted to the women’s hand size [20]. The highest recorded strength values (in kg) were used for analysis.

#### 2.2.5. Physical Activity, Sleep Quality

Participants were instructed to wear ActiGraph GT3X+ devices (ActiGraph LLC, Pensacola, FL, US) attached to their non-dominant wrist for nine consecutive days, 24 h a day. Participants were encouraged to wear the devices during awake and sleep hours and to remove them for water-based activities. The devices were set to collect data at a sample rate of 90 Hz and with the ActiGraph Idle Sleep Mode activated. Once the data collection was finished, the devices were returned to the research facilities. Devices’ data were downloaded using the ActiLife v.6.13.6 software (ActiGraph, Pensacola, FL, USA) in .gt3x files. These files were processed in the GGIR R Package version 3.1.1 [21]. The processing pipeline in GGIR included the following steps: (i) acceleration data were calibrated using the automatic algorithm by van Hees et al. [22]; (ii) non-wear time was detected based on the standard deviation and range of the raw accelerations following the default methods in GGIR, i.e., the updated version of the algorithm by van Hees et al. [23]; (iii) as standard in GGIR, time gaps produced by the ActiGraph Idle Sleep Mode were imputed, and clipping time characterized by unusually sustained high accelerations was detected and classified as invalid; (iv) Euclidean Norm Minus One G with negative values rounded to zero (ENMO) [24], the angle of the z-axis of the device relative to the horizontal plane, and step counts were derived from the raw accelerations. The Verisens step count algorithm with the updated parameters provided by Rowlands et al. [25] was used; (v) for each night in the recording, the sleep period time was detected, defined as the main episode of sleep in the day. The sleep period time was detected using the HDCZA algorithm based on the z-axis angle variability over the day as described in [26]; (vi) the physical activity intensity was classified based on ENMO using the Hildebrand et al. cut-points for adults of 35 mg for light intensity, 100 mg for moderate intensity, and 400 mg for vigorous intensity [27]. Bouts of [30–60) and [60, Inf) min of inactivity, [10, Inf) min of light intensity, and [1–5), [5–10), and [10, Inf) min of moderate-to-vigorous physical activity (MVPA) were identified.

#### 2.2.6. Subjective Evaluations of Health and Wellness

Subjective health and wellness were evaluated through a battery of previously validated questionnaires in athletes to obtain a comprehensive evaluation of their self-reported well-being. Symptoms of androgen deficiency in aging males were assessed using the Androgen Deficiency in Aging Males Questionnaire (ADAM-Q), including factors such as low libido, energy levels, and mood changes [28]. Eating behaviors and attitudes associated with eating disorders were screened using the Eating Attitudes Test-26 (EAT-26), assessing concerns related to dieting, weight, and control [29]. Body image perceptions were assessed using the Silhouette Rating Scale (SRS), which utilizes body silhouette images to evaluate individuals’ attitudes toward their body shape and size [30]. Athlete’s self-perceived performance and personal evaluation of how well they performed in training or competition were evaluated using Athlete’s Subjective Performance Scale (ASPS) [31]. The subjective quality and quantity of sleep were evaluated using the Pittsburgh Sleep Quality Index (PSQI) with higher scores indicating poorer sleep quality [32]. Signs of low energy availability in female athletes were identified using the Low Energy Availability in Females Questionnaire (LEAF-Q), assessing factors such as injuries, gastrointestinal function, and menstrual cycle [33]. The nutritional knowledge of athletes was assessed using the Nutrition Knowledge Questionnaire for Young Athletes (NUKYA), focusing on key areas such as nutrient intake, hydration, and diet for performance [34]. The risk of overtraining by monitoring signs of excessive physical and mental fatigue was evaluated using the Overtraining Score [35]. The balance between stress and recovery in athletes was measured using the Recovery–Stress Questionnaire for Athletes (RESTQ-76), assessing physical, emotional, and social dimensions to identify factors that impact performance and well-being [35].

### 2.3. Statistical Analyses

Unless otherwise indicated, descriptive data are expressed as mean and standard deviation (SD). Data normality was checked using histograms, Q-Q plots, box plots, and the Shapiro–Wilk test. We used one-way analysis of variance (ANOVA) with post hoc Bonferroni correction to compare the multidimensional differences between athletes of endurance, strength, and intermittent sports in body composition, dietary outcomes, RMR, physical activity, sleep quality, and subjective health and wellness. Effect sizes were determined by calculating eta-squared (η^2^) with η^2^ < 0.01 indicating a trivial effect size, 0.01–0.06 a small effect size, 0.06–0.14 a moderate effect size, and η^2^ > 0.14 a large effect size. In addition, we used a one-way analysis of covariance (ANCOVA) to compare the multidimensional differences between athletes of endurance, strength, and intermittent sports in the dependent outcomes adjusting for sex.

Figures were built with GraphPad Prism software v.8 (GraphPad Software, San Diego, CA, USA), and all analyses were performed using the Statistical Package for the Social Sciences v.27.0 (IBM Corporation, Pittsburgh, PA, USA).

## 3. Results

The descriptive characteristics of the study participants are included in Table 1.

### 3.1. Differences in Body Composition Between Endurance, Strength, and Intermittent Sports Athletes

**Figure 1:**
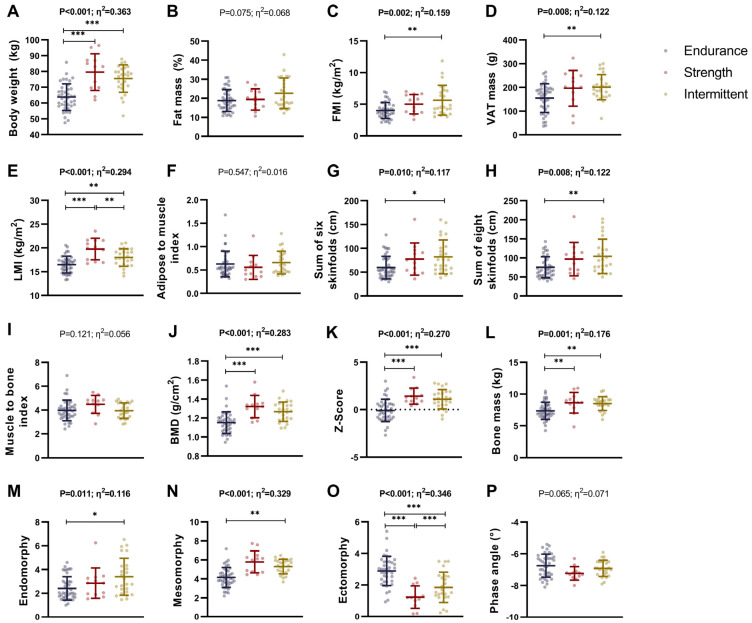
Differences in body composition between endurance, strength, and intermittent sports athletes. Differences in body weight (**A**), adiposity (**B**–**D**), lean mass index (**E**), adipose to muscle index (**F**), sum of skinfolds (**G**,**H**), muscle to bone index (**I**), bone parameters (**J**–**L**), somatotype (**M**–**O**), and phase angle (**P**). Data represent mean and standard deviation. *p* values and η^2^ obtained from one-way analysis of variance (ANOVA). Bold *p* values and η^2^ represent statistically significant *p* values. Symbols mean statistically significant differences between groups after post hoc Bonferroni correction (* = *p* < 0.05; ** = *p* < 0.01; *** = *p* < 0.001). Abbreviations: BMD, bone mineral density; FMI, fat mass index; LMI, lean mass index; VAT, visceral adipose tissue.

We observed that both strength and intermittent athletes displayed higher body weight than endurance athletes (*p* < 0.001; Figure 1A). In addition, intermittent athletes showed higher adipose indices (i.e., FM, FM percentage, FMI, VAT mass, FM android, FM gynoid, adipose mass, the sum of six and eight skinfolds; all *p* ≤ 0.037; Figure 1B–D,G,H and Table S1) than endurance athletes. We also showed that strength athletes displayed higher lean indices (i.e., LM, LMI, FFM, muscle mass) than endurance athletes (all *p* ≤ 0.003; Figure 1E and Table S1). Similarly, intermittent athletes showed higher lean indices (i.e., LM, LMI, FFM, muscle mass) than endurance athletes (all *p* ≤ 0.026; Figure 1E and Table S1). Additionally, we demonstrated that both strength and intermittent sports athletes exhibited higher bone parameters (i.e., BMC, BMD, Z-score, and bone mass) than endurance athletes (all *p* ≤ 0.013; Figure 1J–L and Table S1). Intermittent athletes exhibited greater endomorphy and ectomorphy values than endurance athletes (*p* ≤ 0.008; Figure 1M,N and Table S1). Endurance athletes presented higher values of ectomorphy than both strength and intermittent athletes (both *p* < 0.001; Figure 1O and Table S1), whereas intermittent athletes presented higher ectomorphy values than strength athletes (*p* < 0.001; Figure 1O and Table S1). No differences between endurance, strength, and intermittent athletes were observed in the adipose-to-muscle index, muscle-to-bone index, or phase angle (all *p* > 0.05; Figure 1F,I and Table S1).

These results were similar when we included sex as a covariate in the analyses (Table S1).

### 3.2. Differences in Dietary Intake Between Endurance, Strength, and Intermittent Sports Athletes

**Figure 2:**
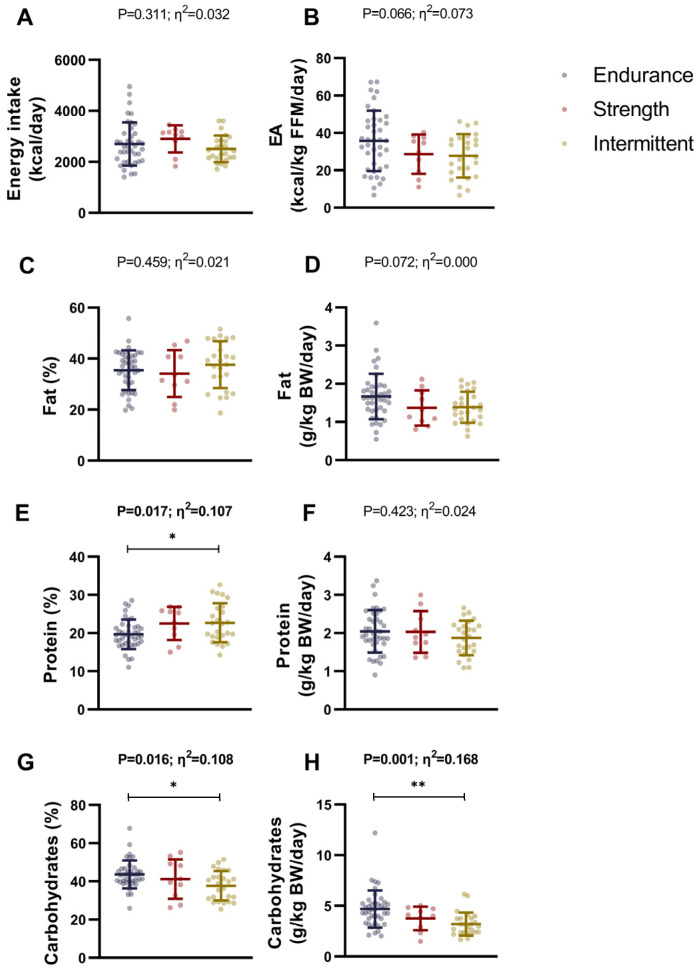
Differences in dietary intake between endurance, strength, and intermittent sports athletes. Differences in energy intake (**A**), energy availability (**B**), fat intake (**C**,**D**), protein intake (**E**,**F**), and carbohydrate intake (**G**,**H**). Data represent mean and standard deviation. *p* values and η^2^ obtained from one-way analysis of variance (ANOVA). Bold *p* values and η^2^ represent statistically significant *p* values. Symbols mean statistically significant differences between groups after post hoc Bonferroni correction (* = *p* < 0.05; ** = *p* < 0.01). Abbreviations: BW, body weight; EA, energy availability; FFM, fat-free mass.

No significant differences were found between endurance, strength, and intermittent athletes for total energy intake (*p* = 0.311; Figure 2A), energy availability (*p* = 0.066; Figure 2B), fat intake percentage (*p* = 0.459; Figure 2C), fat intake normalized by body weight (*p* = 0.072; Figure 2D), or protein intake normalized by body weight (all *p* ≥ 0.066; Figure 2F). However, intermittent athletes showed a significantly higher protein intake percentage than endurance athletes (*p* = 0.025; Figure 2E). Additionally, endurance athletes presented higher carbohydrate intake percentage (*p* = 0.016; Figure 2G), and carbohydrate intake normalized by body weight (*p* = 0.001; Figure 2H) than intermittent athletes. No statistically significant differences were observed between sport discipline groups in sugar and dietary fiber intake, and the consumption of all food groups (all *p* > 0.05; Tables S2 and S3).

These results were similar when we included sex as a covariate in the analyses (Tables S2 and S3).

### 3.3. Differences in Resting Metabolic Rate and Handgrip Between Endurance, Strength, and Intermittent Sports Athletes

**Figure 3:**
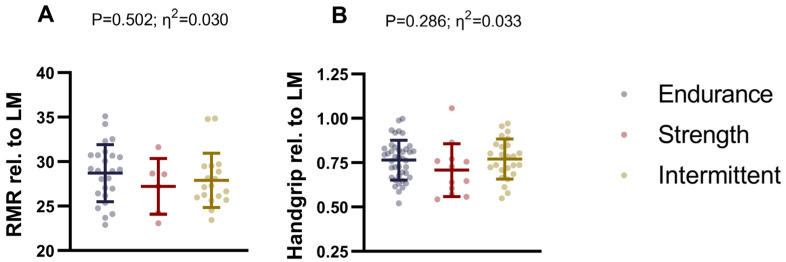
Differences in resting metabolic rate (**A**) and handgrip (**B**) between endurance, strength, and intermittent sports athletes. Resting metabolic rate and handgrip strength were relativized to lean mass. Data represent mean and standard deviation. *p* values and η^2^ obtained from one-way analysis of variance (ANOVA). Abbreviations: LM, lean mass; RMR, resting metabolic rate.

No significant differences were observed in RMR relative to lean mass among endurance, strength, and intermittent athletes (*p* = 0.502; Figure 3A and Table S4). Similarly, handgrip strength relative to lean mass did not differ significantly between the sports discipline groups (*p* = 0.286; Figure 3B and Table S4). These results did not change after including sex as a covariate in the analyses (Table S4).

### 3.4. Differences in Physical Activity and Sleep Quality Between Endurance, Strength, and Intermittent Sports Athletes

**Figure 4:**
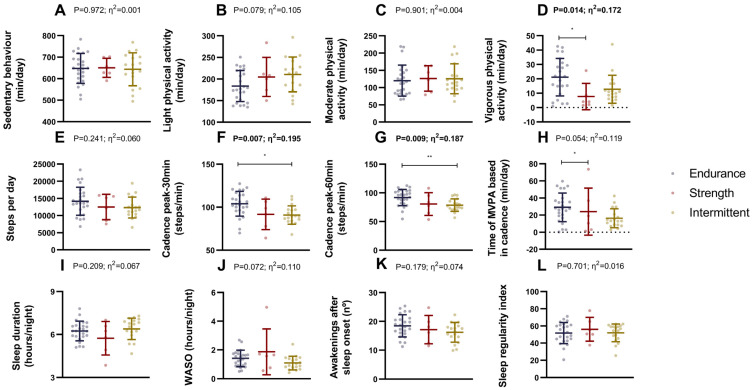
Differences in physical activity and sleep quality between endurance, strength, and intermittent sports athletes. Differences in sedentary behaviour (**A**), physical activity (**B**–**D**), steps (**E**), cadence peak (**F**,**G**), time of MVPA based in cadence (**H**), sleep duration (**I**), and quality (**J**–**L**). Data represent mean and standard deviation. *p* values and η^2^ obtained from one-way analysis of variance (ANOVA). Bold *p* values and η^2^ represent statistically significant *p* values. Symbols mean statistically significant differences between groups after post hoc Bonferroni correction (* = *p* < 0.05; ** = *p* < 0.01). Abbreviations: MVPA, moderate-to-vigorous physical activity; WASO, wake after sleep onset.

No significant differences were observed in sedentary behavior, light physical activity, moderate physical activity, bouts of MVPA (1–5 min, 5–10 min, 10 min), bouts of sedentary behavior (30–60 min, 60 min), or steps per day among endurance, strength, and intermittent athletes (all *p ≥* 0.079, respectively; Figure 4A–C,E and Table S5). However, endurance athletes displayed higher vigorous physical activity levels than strength athletes (*p* = 0.014; Figure 4D and Table S5). Additionally, endurance athletes showed higher cadence peaks during 30 and 60 min than intermittent sports athletes (both *p* ≤ 0.010, respectively; Figure 4F,G and Table S5). No differences between endurance, strength, and intermittent athletes were observed in time spent at 0, 1–19, 20–39, 40–59, 60–79, and 100–119 steps/min (all *p* > 0.06; Table S5). However, endurance athletes spent more time at 80–99 and at ≥120 steps/min than strength athletes (both *p* ≤ 0.026; Table S5). Additionally, endurance athletes spent more time in MVPA based on cadence than strength athletes (*p* = 0.049; Figure 4H and Table S5).

In terms of sleep parameters, no significant differences were observed in sleep duration, wake after sleep onset (WASO), number of awakenings, sleep regularity index, and the sustained inactivity bouts during waking and sleep (nº and duration), between groups (all *p* ≥ 0.05; Figure 4I–L and Table S5).

These results were similar when we included sex as a covariate in the analyses (Table S5).

### 3.5. Differences in Subjective Health and Wellness Between Endurance, Strength, and Intermittent Sports Athletes

No significant differences were observed in the total score of ADAM-Q, EAT-26, SRS, ASPS, PSQI, NUKYA, Overtraining, and RESTQ-76 subscores between endurance, strength, and intermittent sports athletes (all *p* > 0.06; Table 2 and Table S6). Although we observed no differences in LEAF-Q total score, GI function score, and menstrual cycle score (all *p* > 0.05; Table 2 and Table S6), we observed that intermittent athletes displayed higher LEAF-Q injuries score than endurance athletes (*p* = 0.033; Table 2 and Table S6). These results did not change after including sex as a covariate in the analyses (Table S6).

## 4. Discussion

This study identifies distinct physiological phenotypes between strength, intermittent, and endurance athletes. Specifically, strength and intermittent athletes exhibited significantly greater body weight, bone parameters, and lean mass compared to endurance athletes while intermittent athletes showed higher adiposity levels than endurance athletes. Although total energy intake did not differ, endurance athletes consumed more carbohydrates than intermittent athletes, whereas intermittent athletes had a higher protein intake than endurance athletes. Additionally, endurance athletes also engaged in more vigorous physical activity but showed no differences in resting metabolic rate, handgrip strength, sleep parameters, or subjective health measures.

### 4.1. Differences in Body Composition

Body composition (i.e., adipose, lean, and bone) is proposed as a key physiological parameter that predicts performance and health in athletes [36,37]. Our findings indicate that intermittent athletes exhibit higher adiposity levels than endurance athletes. Adiposity in athletes plays a complex physiological role. In endurance sports, low adiposity can improve oxygen efficiency and reduce the mechanical load on the body [38]. However, in strength and intermittent sports, a certain level of body fat can provide cushioning, and protection from impacts, and enhance stability during high-impact actions [39]. Thus, the optimal balance of adiposity varies by sport and its specific demands, as both excessive and insufficient adiposity levels can negatively affect performance and long-term health.

Specifically, lean mass is crucial for athletes, as it is closely related to strength, power, and overall performance while supporting metabolic efficiency and reducing injury risk [40]. A previous study showed endurance athletes had the lowest muscle mass compared to other categories (e.g., team sports, combat sports, CrossFit^®^) [41], which is in line with our results. Similarly, strength and team sports athletes display higher muscle mass and muscular strength than endurance athletes due to specific training adaptations [42]. Lean mass is crucial in strength disciplines and team sports [8], and sports requiring maximal strength and power [43], since these sports rely on anaerobic energy resources for explosive, decisive actions and emphasize strength training to promote muscle hypertrophy [44].

Bone parameters are physiologically essential for athletes, providing structural support, reducing fracture risk, and enhancing resilience to the mechanical demands of intense physical activity [45]. Strength and intermittent athletes exhibit higher bone parameters than endurance athletes, consistent with previous studies showing that strength athletes have greater BMD than endurance athletes due to the intense mechanical loads of their training [46], which stimulates bone remodeling through osteoblast activation [47]. The high-intensity, varied stresses in strength and intermittent training elicit stronger adaptive responses compared to the repetitive, lower-impact loads in endurance sports [48]. Thus, mechanical load type and intensity can be key factors in BMD differences between these athlete groups.

### 4.2. Differences in Dietary Intake

Dietary intake of energy and macronutrients is also relevant for athletes, as it provides energy for training and recovery, supports muscle repair and growth, regulates metabolic processes, and optimizes performance [36]. We observed that while total energy intake was similar across groups, endurance athletes had a higher carbohydrate intake than endurance athletes, whereas intermittent athletes consumed more protein than endurance athletes. Our results differ from a previous study on 553 elite Dutch athletes across endurance, team, and strength sports, which reported only small differences in energy and macronutrient intake between these disciplines [10]. Our cross-sectional assessment of athletes may have captured them at specific phases of their sports seasons, each characterized by distinct training loads and objectives. This variability could account for the observed similarity in energy and protein intake between strength and endurance athletes [49]. However, the higher carbohydrate consumption observed in the endurance group in our study may be attributed to athletes’ evidence-based knowledge indicating that a CHO-rich diet optimizes muscle glycogen stores and enhances endurance exercise performance [50]. Based on the important role of CHO, these findings highlight the relevance of tailoring dietary intake to the specific demands of each sport.

### 4.3. Differences in Resting Metabolic Rate and Handgrip Strength

In this study, no significant differences were observed between sports disciplines in RMR or handgrip strength when adjusted for LM. Previous research comparing RMR among athletes from different sports (i.e., handball, football, cycling, and functional fitness) found that handball players had significantly higher RMR values, while cyclists had significantly lower RMR values compared to the other groups [51]. Given that RMR is closely associated with fat-free mass (FFM) [52], the higher body weight and FFM observed in handball players likely explain their elevated RMR. Handgrip strength is often used in clinical settings as an indicator of overall physical strength and health and is also related to muscle mass quantity and function [53]. We observed no differences between sport disciplines athletes when we accounted for LM in both outcomes.

### 4.4. Differences in Physical Activity Levels and Subjective Health

No major differences among endurance, strength, and intermittent athletes were found in sedentary behavior, physical activity, or sleep patterns (i.e., sleep duration, wakefulness, and regularity). The absence of significant differences in sleep parameters may be attributed to the fact that athletes from different sports were evaluated at varying stages of their competitive season. However, endurance athletes tended to engage in more vigorous activity and achieved higher peak cadences than the other groups. Collegiate athletes (i.e., football and lacrosse) exhibited higher daily physical activity levels than non-athletes [54], spending more energy on moderate and vigorous activities and less time on sedentary activities. The findings suggest that assessing both Non-Exercise Activity Thermogenesis and exercise energy expenditure is essential for accurately evaluating athletes’ energy expenditure. Unfortunately, there is a lack of studies comparing activity levels across different sports, which hamper further comparisons.

Our data also showed no significant differences in subjective evaluations of health and wellness among endurance, strength, and intermittent athletes. While overall scores in areas like gastrointestinal function, recovery, and menstrual cycle were similar, intermittent athletes had slightly higher injury scores than endurance athletes. Others showed that bodybuilding had the lowest injury rates (0.24–1 injury per 1000 h) [55] and team sports like football exceed the injury prevalence rate in comparison to endurance and strength sports [56]. This suggests that although general wellness metrics are consistent across different sports types, intermittent athletes may face unique injury risks, highlighting the need for further research and targeted prevention strategies.

### 4.5. Strengths and Limitations

Our study’s main strengths include evaluating a representative sample of Spanish elite and pre-elite athletes across various sports disciplines and subcategories, using different body composition assessment methods (i.e., BIA, DXA, and anthropometry). This is particularly important, as such comprehensive evaluations are rarely included in most studies. In addition, the data on sleep and physical activity are obtained under real-world living conditions, which ensures the extrapolation to non-laboratory conditions. It is important to highlight that this study involves a multitude of variables to consider within a population group that is particularly difficult to access, providing a complete phenotype of the athletes. On the other hand, several limitations need to be acknowledged. The relatively low sample size does not allow us to analyze the data in women and men separately. Additionally, the relatively low sample size of strength athletes compared to endurance and intermittent athletes may have hindered the statistical power of the study. Moreover, the self-administered nature of the questionnaires completed by participants and the lack of biochemical parameters (e.g., hormonal or metabolic parameters) can dampen the generalization of our study findings. Future studies with larger sample sizes and biochemical determinations are needed to understand the physiological differences between different sports disciplines.

## 5. Conclusions

In conclusion, this study reveals that strength and intermittent athletes exhibit greater body mass, and lean and bone parameters compared to endurance athletes, who, in turn, have distinct dietary intakes characterized by higher carbohydrate consumption. Although no differences were observed in resting metabolic rate, handgrip strength, sleep parameters, or subjective health measures, endurance athletes seem to engage in more vigorous physical activity. These findings underscore the need for developing specific training and nutrition protocols tailored to each athlete type. Further research incorporating biochemical analyses with higher sample sizes is warranted to provide a more comprehensive assessment of health and performance in this population.

## Figures and Tables

**Table 1:** Descriptive parameters.

	Endurance Athletes (n = 40)		Strength Athletes (n = 12)		Intermittent Athletes (n = 25)	
	Mean	SD	Mean	SD	Mean	SD
Age (years)	23.7	6.5	29.3	3.7	25.00	4.9
Sex (n; %)						
Male (n, %)	25	62.5	8	66.6	16	64.0
Female (n, %)	15	37.5	4	33.4	9	36.0
Weight (kg)	64.0	8.4	79.9	11.6	75.8	8.6
Height (cm)	171.6	8.8	174.0	7.1	174.3	8.1
BMI (kg/m^2^)	21.7	1.7	26.3	2.6	24.96	2.4
Training experience (years)	8.8	5.5	6.8	6.0	10.1	5.7
Competition level						
Regional (n,%)	17	42.5	5	41.7	15	60
National (n, %)	22	55	7	58.3	7	28
International (n, %)	1	2.5	0	0	2	8

Data presented as mean and standard deviation (SD), unless otherwise indicated. Abbreviations: BMI, body mass index.

**Table 2:** Differences in subjective health and wellness between endurance, strength, and intermittent sports athletes.

	Endurance Athletes			Strength Athletes			Intermittent Athletes			One-Way ANOVA	
	Mean	SD	n	Mean	SD	n	Mean	SD	n	*p* Value	η^2^
ADAM-Q (Total score)	2.12	2.36	26	2.13	1.55	8	1.63	2.22	16	0.764	0.011
EAT-26 (Total score)	11.56	7.65	39	10.18	5.76	11	12.58	8.47	24	0.687	0.011
SRS (Total score)	−0.18	1.04	38	−0.17	0.72	12	0.04	0.93	25	0.646	0.012
ASPS (Total score)	42.03	10.96	39	34.83	9.65	12	40.96	11.57	25	0.143	0.052
PSQI (Total score)	5.45	2.13	38	6.67	3.73	12	6.87	4.65	23	0.235	0.041
LEAF-Q (Total score)	7.07	6.15	14	4.50	2.65	4	12.22	6.32	9	0.062	0.207
LEAF-Q (Injuries score)	1.64	2.68	14	1.75	3.50	4	4.78	2.44	9	**0.033**	0.247
LEAF-Q (GI function score)	2.79	2.78	14	1.50	0.58	4	3.22	2.99	9	0.571	0.046
LEAF-Q (Menstrual cycle score)	2.64	1.69	14	1.25	1.50	4	4.22	3.67	9	0.136	0.153
NUKYA (Total score)	22.46	6.71	39	23.08	8.85	12	24.40	8.29	25	0.611	0.013
Overtraining (Total score)	8.97	8.67	38	12.50	8.76	10	11.78	11.30	23	0.413	0.026
RESTQ-76—General stress score	2.23	2.19	39	2.30	2.00	10	3.78	6.13	23	0.301	0.034
RESTQ-76—Emotional stress score	3.26	2.83	39	3.90	2.51	10	3.70	4.39	23	0.811	0.006
RESTQ-76—Social stress score	2.82	2.45	39	3.70	2.21	10	3.57	4.83	23	0.615	0.014
RESTQ-76—Conflicts/pressure score	6.56	3.65	39	6.80	5.88	10	6.52	5.31	23	0.986	0.000
RESTQ-76—Fatigue score	6.77	3.14	39	6.50	3.54	10	8.04	6.12	23	0.479	0.021
RESTQ-76—Lack of energy score	4.69	3.12	39	5.00	3.40	10	5.39	4.49	23	0.767	0.008
RESTQ-76—Physical alterations score	3.95	2.76	39	3.10	1.37	10	4.43	4.19	23	0.538	0.018
RESTQ-76—Success score	11.00	5.32	39	11.70	4.60	10	11.13	5.11	23	0.929	0.002
RESTQ-76—Social recovery score	13.23	4.74	39	11.90	4.79	10	13.57	5.94	23	0.690	0.011
RESTQ-76—Physical recovery score	12.10	4.69	39	10.60	3.24	10	12.48	5.45	23	0.579	0.016
RESTQ-76—General well-being score	16.10	4.99	39	13.90	5.15	10	15.61	6.39	23	0.531	0.018
RESTQ-76—Sleep Quality score	10.44	4.44	39	10.90	4.58	10	10.52	4.25	23	0.957	0.001
RESTQ-76—Alterations of rest periods score	4.33	3.43	39	4.30	2.63	10	3.61	3.00	23	0.676	0.011
RESTQ-76—Emotional fatigue score	3.56	4.27	39	2.40	1.84	10	4.30	3.61	23	0.419	0.025
RESTQ-76—Injuries score	6.23	4.31	39	8.20	4.66	10	7.13	4.35	23	0.407	0.026
RESTQ-76—Being in shape score	13.64	5.20	39	13.50	5.68	10	13.48	5.46	23	0.992	0.000
RESTQ-76—Personal fulfillment score	11.28	5.25	39	12.90	5.02	10	11.74	5.45	23	0.687	0.011
RESTQ-76—Self-efficacy score	12.95	5.31	39	14.10	6.24	10	12.30	5.93	23	0.702	0.010
RESTQ-76—Self-regulation score	14.21	6.36	39	14.70	7.39	10	13.96	5.94	23	0.954	0.001

Data represent mean and standard deviation. *p* values and η^2^ obtained from one-way analysis of variance (ANOVA). Bold *p* values represent statistically significant *p* values. Abbreviations: ADAM-Q, Androgen Deficiency in the Aging Male Questionnaire; ASPS, Athlete’s Subjective Performance Scale; EAT-26, Eating Attitudes Test-26; GI, gastrointestinal; LEAF-Q, Low Energy Availability in Females Questionnaire; NUKYA, Nutrition Knowledge for Young and Adult Athletes; PSQI, Pittsburgh Sleep Quality Index; RESTQ-76, Recovery–Stress Questionnaire for Athletes-76; SRS, Silhouette Rating Scale.

## Data Availability

The data are not publicly available due to privacy reasons. The data supporting this study’s findings are available from the corresponding author, L.J.-F., upon reasonable request.

## Supplementary Materials

The following supporting information can be downloaded at: https://www.mdpi.com/article/10.3390/nu17071172/s1, Table S1. Differences in body composition between endurance, strength, and intermittent sports athletes. Table S2. Differences in dietary intake between endurance, strength, and intermittent sports athletes. Table S3. Differences in food group consumption between endurance, strength, and intermittent sports athletes. Table S4. Differences in resting metabolic rate and handgrip strength between endurance, strength, and intermittent sports athletes. Table S5. Differences in physical activity and sleep quality between endurance, strength, and intermittent sports athletes. Table S6. Differences in subjective health and wellness between endurance, strength, and intermittent sports athletes.
