# Supplementary material for: Multidimensional Differences Between Athletes of Endurance, Strength, and Intermittent Sports: Body Composition, Diet, Resting Metabolic Rate, Physical Activity, Sleep Quality, and Subjective Well-Being
Source: Nutrients. 2025 Mar 28;17(7):1172. doi: 10.3390/nu17071172 (PMC11990401; doi:10.3390/nu17071172)
Supplement: Supplementary file 1 [file nutrients-17-01172-s001.zip › nutrients-3476836-supplementary.pdf]

SUPPLEMENTARY MATERIAL

Table S1. Differences in body composition between endurance, strength, and intermittent sports athletes.

	Endurance athletes			Strength athletes			Intermittent athletes				Effect size			Endurance vs. strength				Endurance vs. intermittent				Strength vs. intermittent				ANCOVA
	Mean	SD	n	Mean	SD	n	Mean	SD	n	Global P value	η²	95% CI		Mean difference	95% CI		P value	Mean difference	95% CI		P value	Mean difference	95% CI		P value	P value
DXA outcomes																										
BMC (g)	2344.71	452.21	40	2955.29	508.90	12	2851.69	470.93	25	**<0.001**	0.261	0.095	0.399	-610.59	-987.21	-233.96	**<0.001**	-506.98	-798.71	-215.25	**<0.001**	103.60	-298.25	505.46	1.000	**<0.001**
BMD (g/cm^2)	1.15	0.11	40	1.32	0.12	12	1.27	0.10	25	**<0.001**	0.283	0.112	0.419	-0.17	-0.26	-0.08	**<0.001**	-0.12	-0.18	-0.05	**<0.001**	0.05	-0.04	0.15	0.515	**<0.001**
Z-score	-0.09	1.18	40	1.42	0.85	12	1.09	1.02	25	**<0.001**	0.270	0.102	0.407	-1.5017	-2.378	-0.625	**<0.001**	-1.173	-1.852	-0.494	**<0.001**	0.3287	-0.607	1.264	1.000	**<0.001**
LM (kg)	48.88	8.51	40	60.17	9.65	12	54.89	8.72	25	**<0.001**	0.195	0.048	0.333	-11.29	-18.35	-4.23	**0.001**	-6.01	-11.48	-0.54	**0.026**	5.28	-2.26	12.81	0.271	**<0.001**
LMI (kg/m^2)	16.49	1.76	40	19.77	2.25	12	17.97	1.86	25	**<0.001**	0.294	0.122	0.430	-3.28	-4.79	-1.77	**<0.001**	-1.48	-2.65	-0.31	**0.008**	1.80	0.19	3.41	**0.023**	**<0.001**
FFM (kg)	51.22	8.86	40	63.12	10.08	12	57.74	9.09	25	**<0.001**	0.201	0.051	0.339	-11.90	-19.26	-4.54	**0.001**	-6.52	-12.22	-0.82	**0.019**	5.38	0.29	-2.47	0.292	**<0.001**
FM (kg)	11.65	3.12	40	15.18	4.74	12	16.83	6.28	25	**<0.001**	0.218	0.063	0.356	-3.54	-7.25	0.18	0.067	-5.19	-8.07	-2.31	**<0.001**	-1.65	-5.62	2.32	0.934	**<0.001**
FM (%)	18.84	5.72	40	19.39	5.57	12	22.63	8.01	25	0.075	0.068	0.000	0.182	-0.55	-5.82	4.71	1.000	-3.79	-7.86	0.29	0.078	-3.23	-8.85	2.38	0.488	**0.002**
FMI (kg/m^2)	4.02	1.26	40	5.01	1.53	12	5.64	2.36	25	**0.002**	0.159	0.027	0.295	-0.99	-2.39	0.40	0.258	-1.63	-2.71	-0.55	**0.001**	-0.64	-2.13	0.86	0.900	**<0.001**
FM android	672.28	177.34	40	898.98	341.05	12	965.20	381.32	25	**<0.001**	0.195	0.048	0.333	-226.69	-456.19	2.81	0.054	-292.92	-470.69	-115.15	**<0.001**	-66.23	-311.10	178.65	1.000	**<0.001**
FM ginoid	2.17	0.73	40	2.86	0.95	12	3.20	1.41	25	**0.001**	0.178	0.038	0.316	-0.68	-1.51	0.15	0.144	-1.03	-1.67	-0.38	**0.001**	-0.35	-1.23	0.54	1.000	**<0.001**
VAT mass (g)	155.04	60.64	40	196.20	75.54	12	201.03	52.94	25	**0.008**	0.122	0.010	0.253	-41.16	-90.17	7.85	0.130	-45.99	-83.96	-8.03	**0.012**	-4.83	-57.13	47.47	1.000	**0.002**
BIA outcomes																										
Weight (kg)	63.75	8.42	40	79.64	11.60	12	75.50	8.65	25	**<0.001**	0.363	0.183	0.491	-15.90	-23.18	-8.61	**<0.001**	-11.75	-17.40	-6.11	**<0.001**	4.14	-3.63	11.91	0.588	**<0.001**
BMI (kg/m^2)	21.61	1.69	40	26.18	2.61	12	24.85	2.33	25	**<0.001**	0.464	0.287	0.578	-4.58	-6.25	-2.91	**<0.001**	-3.25	-4.54	-1.95	**<0.001**	1.33	-0.45	3.11	0.213	**<0.001**
FM (kg)	8.48	3.24	40	14.00	4.43	12	13.45	6.31	25	**<0.001**	0.245	0.082	0.383	-5.52	-9.24	-1.79	**0.002**	-4.97	-7.86	-2.08	**<0.001**	0.55	-3.43	4.52	1.000	**<0.001**
FM (%)	13.60	5.74	40	17.51	4.72	12	17.74	7.80	25	**0.023**	0.097	0.001	0.221	-3.91	-9.04	1.21	0.196	-4.15	-8.12	-0.18	**0.037**	-0.24	-5.70	5.23	1.000	**<0.001**
FFM (kg)	55.26	9.14	40	65.64	9.86	12	62.05	8.93	25	**0.001**	0.174	0.035	0.311	-10.38	-17.79	-2.97	**0.003**	-6.79	-12.52	-1.05	**0.015**	3.59	-4.31	11.50	0.807	**<0.001**
Muscle mass (kg)	52.49	8.71	40	62.38	9.42	12	58.95	8.51	25	**0.001**	0.174	0.035	0.311	-9.90	-16.96	-2.84	**0.003**	-6.47	-11.94	-1.00	**0.015**	3.43	-4.10	10.97	0.805	**<0.001**
Phase angle (º)	-6.75	0.73	40	-7.23	0.43	12	-6.92	0.51	25	0.065	0.071	0.000	0.187	0.48	-0.02	0.99	0.064	0.17	-0.22	0.56	0.901	-0.32	-0.85	0.22	0.456	0.051

Anthropometry outcomes																										
Adipose mass (kg)	16.68	3.20	40	19.88	5.33	12	20.71	5.47	25	**0.001**	0.162	0.029	0.298	-3.20	-6.75	0.35	0.090	-4.02	-6.77	-1.28	**0.002**	-0.82	-4.61	2.96	1.000	**<0.001**
Muscle mass (kg)	28.86	6.65	40	38.16	7.71	12	33.04	6.98	25	**<0.001**	0.197	0.049	0.335	-9.31	-14.89	-3.72	**<0.001**	-4.18	-8.51	0.14	0.061	5.12	-0.83	11.08	0.116	**<0.001**
Bone mass (kg)	7.35	1.36	40	8.64	1.63	12	8.51	1.07	24	**0.001**	0.176	0.036	0.314	-1.28	-2.35	-0.22	**0.013**	-1.15	-1.99	-0.32	**0.004**	0.13	-1.02	1.27	1.000	**<0.001**
Residual mass (kg)	7.25	1.54	40	9.05	1.90	12	8.35	1.21	25	**0.001**	0.186	0.042	0.323	-1.80	-3.01	-0.59	**0.001**	-1.10	-2.04	-0.17	**0.015**	1.10	0.17	2.04	0.015	**<0.001**
Sum of six skinfolds (cm)	59.72	23.58	40	77.51	33.98	12	82.04	35.50	25	**0.010**	0.117	0.007	0.246	-17.79	-41.62	6.03	0.214	-22.33	-40.78	-3.87	**0.012**	-4.54	-29.96	20.89	1.000	**<0.001**
Sum of eight skinfolds (cm)	75.32	27.55	40	96.87	43.94	12	104.10	45.16	25	**0.008**	0.122	0.009	0.252	-21.55	-51.16	8.06	0.236	-28.78	-51.72	-5.85	**0.009**	-7.23	-38.82	24.36	1.000	**<0.001**
Corrected arm circumference (cm)	24.88	3.50	40	30.65	3.66	12	27.10	3.05	25	**<0.001**	0.275	0.105	0.411	-5.77	-8.50	-3.04	**<0.001**	-2.22	-4.34	-0.11	**0.036**	3.55	0.64	6.46	0.012	**<0.001**
Corrected chest circumference (cm)	87.19	6.88	40	96.30	7.68	12	90.81	5.90	25	**<0.001**	0.195	0.048	0.333	-9.11	-14.52	-3.70	**<0.001**	-3.63	-7.82	0.57	0.112	5.48	-0.29	11.26	0.068	**<0.001**
Corrected thigh circumference (cm)	45.86	4.78	40	51.01	4.39	12	48.83	4.12	25	**0.001**	0.165	0.030	0.301	-5.15	-8.79	-1.51	**0.003**	-2.97	-5.79	-0.15	**0.036**	2.18	-1.70	6.07	0.519	**<0.001**
Corrected calf (cm)	32.22	2.83	40	33.21	2.92	12	34.19	2.89	25	**0.030**	0.090	0.000	0.213	-0.99	-3.30	1.32	0.895	-1.97	-3.76	-0.18	**0.026**	-0.98	-3.45	1.48	0.995	**0.003**
Adipose to muscle index	0.63	0.28	40	0.56	0.26	12	0.66	0.24	25	0.547	0.016	0.000	0.091	0.07	-0.14	0.28	1.000	-0.03	-0.19	0.14	1.000	-0.10	-0.33	0.12	0.823	0.362
Muscle-to-bone index	3.97	0.86	40	4.48	0.75	12	3.95	0.66	24	0.121	0.056	0.000	0.166	-0.51	-1.14	0.12	0.158	0.01	-0.48	0.51	1.000	0.52	-0.16	1.21	0.189	0.099
Endomorphy	2.41	0.98	40	2.86	1.27	12	3.39	1.55	25	**0.011**	0.116	0.007	0.245	-0.44	-1.44	0.55	0.838	-0.98	-1.75	-0.21	**0.008**	-0.54	-1.60	0.53	0.665	**<0.001**
Mesomorphy	4.15	1.04	40	5.79	1.15	12	5.29	0.77	24	**<0.001**	0.329	0.150	0.462	-1.64	-2.44	-0.85	**<0.001**	-1.15	-1.77	-0.52	**<0.001**	0.50	-0.36	1.35	0.476	**<0.001**
Ectomorphy	2.89	0.93	40	1.24	0.71	12	1.85	0.96	25	**<0.001**	0.346	0.167	0.477	1.66	0.92	2.39	**<0.001**	1.04	0.47	1.60	**<0.001**	-0.62	-2.39	-0.92	**<0.001**	**<0.001**

Data represent mean and standard deviation, mean difference, and 95% confidence interval. Global P values and η^2 obtained from one-way analysis of variance (ANOVA) and between groups differences P values obtained after post-hoc Bonferroni correction. P values for the analysis of covariance (ANCOVA) adjusted for sex are also shown. Bold values mean statistically significant P values. *Abbreviations*: BIA, bioimpedance; BMC, bone mineral content; BMD, bone mineral density; BMI, body mass index; CI, confidence interval; DXA, Dual-Energy X-ray Absorptiometry; ECW, extracellular water; FFM, fat-free mass; FM, fat mass; FMI, fat mass index; ICW, intracellular water; LM, lean mass; LMI, lean mass index; TBW, total body water; VAT, visceral adipose tissue.

Table S2. Differences in dietary intake between endurance, strength, and intermittent sports athletes.

	**Endurance athletes**			**Stregnth athletes**			**Intermittent athletes**				**Effect size**			**Endurance vs. strength**				**Endurance vs. intermittent**				**Strength vs. intermittent**				**ANCOVA**
	Mean	SD	n	Mean	SD	n	Mean	SD	n	P value	η^2	95% CI		Mean difference	95% CI		P value	Mean difference	95% CI		P value	Mean difference	95% CI		P value	P value
Energy intake (kcal/day)	2698.75	844.05	40	2899.22	526.80	10	2507.47	523.46	25	0.311	0.032	0.000	0.126	-200.47	-820.53	419.60	1.000	191.28	-255.85	638.42	0.894	391.75	-264.46	1047.96	0.443	0.305
EA (kcal/kg FFM/day)	35.73	16.15	40	28.69	10.50	10	27.70	11.63	25	0.066	0.073	0.000	0.191	7.05	-5.21	19.31	0.489	8.04	-0.80	16.88	0.087	0.99	-11.98	13.96	1.000	0.065
Fat (g/day)	106.05	42.27	40	108.47	31.74	10	104.75	33.10	25	0.966	0.001	0.000	0.008	-2.42	-35.52	30.69	1.000	1.30	-22.57	25.18	1.000	3.72	-31.31	38.76	1.000	0.978
Fat (%)	35.44	7.81	40	34.12	9.20	10	37.62	9.23	25	0.459	0.021	0.000	0.104	1.32	-6.03	8.67	1.000	-2.18	-7.48	3.12	0.951	-3.50	-11.28	4.28	0.822	0.468
Fat (g/kg body weight/day)	1.67	0.60	40	1.37	0.46	10	1.39	0.41	25	0.072	0.000	0.190	0.245	0.30	-0.16	0.75	0.334	0.28	-0.05	0.61	0.117	-0.02	-0.50	0.46	1.000	0.068
SFA (g/day)	27.23	14.51	40	28.33	13.14	10	26.95	11.24	25	0.962	0.001	0.000	0.011	-1.10	-12.65	10.46	1.000	0.29	-8.05	8.62	1.000	1.38	-10.84	13.61	1.000	0.973
MUFA (g/day)	37.58	16.07	40	36.92	14.49	10	38.90	14.88	25	0.923	0.002	0.000	0.030	0.66	-12.77	14.08	1.000	-1.32	-11.00	8.36	1.000	-1.98	-16.18	12.23	1.000	0.895
PUFA (g/day)	16.20	8.08	40	15.30	5.30	10	16.86	6.94	25	0.847	0.005	0.000	0.050	0.91	-5.51	7.33	1.000	-0.66	-5.29	3.97	1.000	-1.56	-8.36	5.23	1.000	0.845
Cholesterol (mg/day)	421.75	208.02	40	621.68	164.15	9	526.57	232.03	25	**0.020**	0.104	0.002	0.233	-199.94	-391.85	-8.02	**0.038**	-104.82	-237.44	27.80	0.170	95.12	-107.09	297.33	0.758	**0.022**
Protein (g/day)	129.99	39.92	40	160.56	33.86	10	139.54	31.24	25	0.063	0.074	0.000	0.193	-30.57	-62.19	1.06	0.062	-9.55	-32.36	13.25	0.924	21.01	-12.46	54.48	0.385	0.071
Protein (%)	19.66	3.87	40	22.53	4.36	10	22.71	5.13	25	**0.017**	0.108	0.003	0.237	-2.87	-6.67	0.94	0.206	-3.04	-5.79	-0.30	**0.025**	-0.18	-4.20	3.85	1.000	**0.015**
Protein (g/kg body weight/day)	2.04	0.56	40	2.03	0.54	10	1.87	0.45	25	0.423	0.024	0.000	0.109	0.02	-0.44	0.47	1.000	0.17	-0.16	0.50	0.605	0.16	-0.32	0.64	1.000	0.428
Carbohydrates (g/day)	297.89	119.83	40	303.14	108.59	10	238.92	80.86	25	0.079	0.068	0.000	0.185	-5.25	-97.90	87.41	1.000	58.96	-7.85	125.78	0.102	64.21	-33.85	162.27	0.339	0.062
Carbohydrates (%)	43.62	7.28	40	41.15	10.28	10	37.72	7.69	25	**0.016**	0.108	0.003	0.237	2.48	-4.33	9.28	1.000	5.91	1.00	10.81	**0.013**	3.43	-3.77	10.63	0.740	**0.015**
Carbohydrates (g/kg body weight/day)	4.69	1.83	40	3.76	1.17	10	3.20	1.14	25	**0.001**	0.168	0.030	0.306	0.93	-0.42	2.28	0.290	1.49	0.52	2.46	**0.001**	0.56	-0.86	1.99	1.000	**0.001**
Sugars (g/day)	0.70	1.14	40	0.29	0.62	10	0.49	0.89	25	0.458	0.021	0.000	0.104	0.41	-0.465	1.281	0.768	0.21	-0.421	0.838	1.000	-0.20	-1.123	0.725	1.000	0.447
Dietary fiber (g/day)	0.31	0.74	40	0.85	1.47	10	0.41	0.67	25	0.206	0.043	0.000	0.146	-0.54	-1.274	0.196	0.229	-0.10	-0.634	0.426	1.000	0.44	-0.343	1.213	0.523	0.226

Data represent mean and standard deviation, mean difference, and 95% confidence interval. Global P values and η^2 obtained from one-way analysis of variance (ANOVA) and between groups differences P values obtained after post-hoc Bonferroni correction. P values for the analysis of covariance (ANCOVA) adjusted for sex are also shown. Bold values mean statistically significant P values. *Abbreviations*: CI, confidence interval; EA, energy availability; FFM, fat-free mass; MUFA, monounsaturated fatty acids; PUFA, polyunsaturated fatty acids; SFA, saturated fatty acids.

Table S3. Differences in food group consumption between endurance, strength, and intermittent sports athletes.

	**Endurance athletes**			**Strength athletes**			**Intermittent athletes**				**Effect size**			**Endurance vs. strength**				**Endurance vs. intermittent**				**Strength vs. intermittent**				**ANCOVA**
	Mean	SD	n	Mean	SD	n	Mean	SD	n	P value	η^2	95% CI		Mean difference	95% CI		P value	Mean difference	95% CI		P value	Mean difference	95% CI		P value	P value
Fruits (portions/day)	1.70	1.08	40	1.83	1.08	10	1.61	1.04	25	0.850	0.005	0.000	0.049	-0.12	-1.049	0.801	1.000	0.10	-0.570	0.764	1.000	0.22	-0.758	1.200	1.000	0.862
Vegetables (portions/day)	1.15	0.87	40	1.47	0.95	10	1.25	0.70	25	0.549	0.017	0.000	0.093	-0.32	-1.038	0.401	0.845	-0.10	-0.619	0.419	1.000	0.22	-0.543	0.980	1.000	0.554
Legumes (portions/week)	1.87	1.34	40	3.93	6.23	10	2.78	4.36	25	0.215	0.042	0.000	0.144	-2.06	-5.086	0.960	0.296	-0.91	-3.090	1.270	0.929	1.15	-2.046	4.353	1.000	0.231
Tubers (portions/week)	2.71	1.94	40	2.58	2.00	10	3.24	1.45	25	0.445	0.022	0.000	0.106	0.13	-1.431	1.687	1.000	-0.53	-1.656	0.593	0.751	-0.66	-2.310	0.991	0.992	0.451
Pasta (portions/week)	3.91	2.38	40	3.74	1.61	10	2.81	2.26	25	0.160	0.050	0.000	0.157	0.17	-1.784	2.124	1.000	1.10	-0.310	2.508	0.179	0.93	-1.138	2.998	0.823	0.164
Rice (portions/week)	3.37	3.10	40	2.48	1.42	10	2.18	1.61	25	0.162	0.049	0.000	0.156	0.90	-1.283	3.075	0.951	1.20	-0.376	2.767	0.199	0.30	-2.007	2.606	1.000	0.170
Bread (portions/week)	3.90	3.20	40	3.14	1.73	10	4.48	2.73	25	0.448	0.022	0.000	0.106	0.76	-1.753	3.272	1.000	-0.58	-2.396	1.227	1.000	-1.34	-4.003	1.315	0.658	0.406
Whole grains (portions/week)	4.25	4.74	40	6.79	4.07	10	3.67	3.49	25	0.150	0.051	0.000	0.159	-2.54	-6.245	1.170	0.293	0.58	-2.089	3.258	1.000	3.12	-0.801	7.045	0.165	0.164
White meat (portions/week)	4.40	3.26	40	4.59	3.62	10	4.92	4.15	25	0.852	0.004	0.000	0.049	-0.19	-3.337	2.948	1.000	-0.52	-2.791	1.741	1.000	-0.33	-3.656	2.995	1.000	0.857
Egg (portions/week)	4.17	2.82	40	4.38	2.60	10	4.36	2.17	25	0.950	0.001	0.000	0.019	-0.21	-2.460	2.040	1.000	-0.18	-1.808	1.438	1.000	0.03	-2.356	2.407	1.000	0.919
Fish (portions/week)	2.51	1.85	40	1.58	1.18	10	2.20	1.58	25	0.291	0.034	0.000	0.129	0.93	-0.534	2.392	0.372	0.31	-0.747	1.363	1.000	-0.62	-2.170	0.927	0.985	0.282
Red meat (portions/week)	1.71	2.19	40	1.79	1.32	10	2.51	2.91	25	0.410	0.024	0.000	0.111	-0.07	-2.131	1.984	1.000	-0.79	-2.278	0.689	0.580	-0.72	-2.898	1.456	1.000	0.405
Nuts (portions/week)	3.41	3.28	40	2.18	1.98	10	3.61	3.91	25	0.510	0.019	0.000	0.098	1.23	-1.695	4.159	0.917	-0.20	-2.313	1.907	1.000	-1.44	-4.532	1.662	0.780	0.521
Soft drinks (portions/week)	0.62	0.95	40	1.49	3.24	10	0.67	0.92	25	0.224	0.041	0.000	0.142	-0.88	-2.127	0.377	0.273	-0.05	-0.956	0.850	1.000	0.82	-0.503	2.147	0.398	0.242
Sweets, pastries, and candies (portions/week)	2.41	3.71	40	0.84	1.73	10	1.35	2.17	25	0.219	0.041	0.000	0.143	1.57	-1.085	4.228	0.454	1.06	-0.851	2.980	0.532	-0.51	-3.318	2.305	1.000	0.213
Processed foods (portions/week)	0.70	1.14	40	0.29	0.62	10	0.49	0.89	25	0.458	0.021	0.000	0.104	0.41	-0.465	1.281	0.768	0.21	-0.421	0.838	1.000	-0.20	-1.123	0.725	1.000	0.447
Alcoholic drinks (portions/week)	0.31	0.74	40	0.85	1.47	10	0.41	0.67	25	0.206	0.043	0.000	0.146	-0.54	-1.274	0.196	0.229	-0.10	-0.634	0.426	1.000	0.44	-0.343	1.213	0.523	0.226

Data represent mean and standard deviation, mean difference, and 95% confidence interval. Global P values and η^2 obtained from one-way analysis of variance (ANOVA) and between groups differences P values obtained after post-hoc Bonferroni correction. P values for the analysis of covariance (ANCOVA) adjusted for sex are also shown. Bold values mean statistically significant P values. *Abbreviations*: CI, confidence interval.

Table S4. Differences in resting metabolic rate and handgrip strength between endurance, strength, and intermittent sports athletes.

	Endurance athletes			Strength athletes			Intermittent athletes				Effect size			Endurance vs. strength				Endurance vs. intermittent				Strength vs. intermittent				ANCOVA
	Mean	SD	n	Mean	SD	n	Mean	SD	n	P value	η^2	95% CI		Mean difference	95% CI		P value	Mean difference	95% CI		P value	Mean difference	95% CI		P value	P value
RMR (kcal/day)	1378.02	290.51	25	1697.11	141.58	6	1599.64	214.17	18	**0.004**	0.212	0.027	0.380	-319.09	-602.97	-35.21	**0.023**	-221.62	-414.66	-28.59	**0.019**	97.47	-196.90	391.84	1.000	**0.006**
RMR rel. to LM	28.71	3.21	25	27.23	3.14	6	27.89	3.05	18	0.502	0.030	0.000	0.144	1.48	-2.07	5.04	0.916	0.82	-1.60	3.23	1.000	-0.67	-4.35	3.02	1.000	0.667
Handgrip (kg)	37.38	8.50	40	43.15	13.63	12	42.50	10.24	25	0.070	0.069	0.000	0.185	-5.77	-13.82	2.28	0.250	-5.12	-11.36	1.12	0.144	0.65	-7.94	9.24	1.000	**0.013**
Handgrip rel. to LM	0.76	0.11	40	0.71	0.15	12	0.77	0.11	25	0.286	0.033	0.000	0.127	0.06	-0.04	0.15	0.450	-0.01	-0.08	0.07	1.000	-0.06	-0.16	0.04	0.411	0.247

Data represent mean and standard deviation, mean difference, and 95% confidence interval. Global P values and η^2 obtained from one-way analysis of variance (ANOVA) and between groups differences P values obtained after post-hoc Bonferroni correction. P values for the analysis of covariance (ANCOVA) adjusted for sex are also shown. Bold values mean statistically significant P values. *Abbreviations*: CI, confidence interval; LM, lean mass; RMR, resting metabolic rate.

Table S5. Differences in physical activity and sleep quality between endurance, strength, and intermittent sports athletes.

	Endurance athletes			Strength athletes			Intermittent athletes				Effect size			Endurance vs. strength				Endurance vs. intermittent				Strength vs. intermittent				ANCOVA
	Mean	SD	n	Mean	SD	n	Mean	SD	n	P value	η^2	95% CI		Mean difference	95% CI		P value	Mean difference	95% CI		P value	Mean difference	95% CI		P value	P value
Sedentary behaviour (min/day)	647.49	69.47	25	650.48	44.28	6	643.52	76.57	18	0.972	0.001	0.000	0.005	-2.99	-82.04	76.06	1.000	3.97	-49.78	57.73	1.000	6.97	-75.00	88.94	1.000	0.954
Light physical activity (min/day)	183.79	35.80	25	204.80	45.31	6	210.62	40.44	18	0.079	0.105	0.000	0.261	-21.01	-64.70	22.68	0.715	-26.83	-56.54	2.88	0.089	-5.82	-51.12510	39.49	1.000	0.113
Moderate physical activity (min/day)	120.04	44.97	25	126.33	36.98	6	125.50	43.48	18	0.901	0.004	0.000	0.056	-6.28	-55.55	42.98	1.000	-5.45	-39.0	28.05	1.000	0.823	-50.26	51.92	1.000	0.975
Vigorous physical activity (min/day)	21.15	13.1	25	7.68	9.18	6	12.74	9.74	18	**0.014**	0.172	0.007	0.340	13.47	0.41	26.53	**0.041**	8.41	-0.51	17.34	0.070	-5.06	-27.05	16.93	1.000	**0.028**
10min bouts of MVPA (nº)	3.37	1.77	25	2.48	1.33	6	2.60	1.52	18	0.238	0.060	0.000	0.200	0.90	-0.96	2.74	0.708	0.77	-0.49	2.03	0.403	-0.12	-2.04	1.80	1.000	0.166
5-10min bouts of MVPA (nº)	2.39	1.06	25	2.48	1.58	6	2.67	1.21	18	0.733	0.013	0.000	0.102	-0.09	-1.43	1.24	1.000	-0.29	-1.20	0.62	0.366	0.19	-1.19	1.58	1.000	0.612
1-5min bouts of MVPA (nº)	15.04	6.31	25	20.13	13.19	6	22.84	13.66	18	0.059	0.116	0.000	0.275	-5.09	-16.86	6.68	0.865	-7.80	-15.80	0.21	0.059	-2.71	-14.91	9.50	1.000	0.078
60min bouts of sedentary behavior (nº)	3.74	0.97	25	3.53	1.31	6	3.21	1.31	18	0.345	0.045	0.000	0.175	0.20	-1.09	1.50	1.000	0.52	-0.36	1.40	0.440	0.32	-1.02	1.66	1.000	0.400
30-60min bouts of sedentary behavior (nº)	2.94	0.87	25	3.21	1.57	6	2.85	1.01	18	0.767	0.011	0.000	0.095	-0.27	-1.42	0.88	1.000	0.08	-0.70	0.87	1.000	0.35	-0.84	1.55	1.000	0.647
Steps per day	14154.37	4088.96	25	12473.01	3707.09	6	12306.60	3054.68	18	0.241	0.060	0.000	0.199	1681.36	-2494.25	5856.97	0.967	1847.77	-991.54	4687.08	0.338	166.41	-4163.49	4496.32	1.000	0.249
Cadence peak-1min (steps/min)	123.24	14.27	25	114.90	12.32	6	115.70	6.76	18	0.082	0.103	0.000	0.259	8.34	-5.01	21.68	0.382	7.55	-1.53	16.62	0.133	-0.79	-14.63	13.05	1.000	0.164
Cadence peak-30min (steps/min)	104.04	14.46	25	91.67	17.77	6	90.86	10.69	18	**0.007**	0.195	0.018	0.363	12.37	-3.02	27.76	0.155	13.19	2.72	23.65	**0.009**	0.81	-15.14	16.77	1.000	**0.014**
Cadence peak-60min (steps/min)	91.87	14.20	25	80.52	19.87	6	78.57	10.85	18	**0.009**	0.187	0.015	0.354	11.35	-4.29	26.99	0.233	13.30	2.67	23.93	**0.010**	1.95	-14.27	18.16	1.000	**0.017**
Time at 0 steps/min (min/day)	1011.12	89.96	25	989.98	97.21	6	975.17	96.89	18	0.461	0.033	0.000	0.152	21.14	-84.33	126.62	1.000	35.95	-35.77	107.67	0.658	14.81	-94.57	124.18	1.000	0.568
Time at 1-19 steps/min (min/day)	220.07	50.06	25	239.94	48.73	6	254.26	46.80	18	0.084	0.102	0.000	0.258	-19.87	-74.92	35.18	1.000	-34.19	-71.62	3.24	0.084	-14.32	-71.40	42.77	1.000	0.123
Time at 20-39 steps/min (min/day)	78.91	26.42	25	102.39	43.72	6	103.53	41.95	18	0.061	0.114	0.000	0.273	-23.48	-62.97	16.01	0.439	-24.62	-51.47	2.24	0.082	-1.13	-42.09	39.82	1.000	0.092
Time at 40-59 steps/min (min/day)	38.33	16.52	25	47.69	30.07	6	49.63	24.06	18	0.214	0.065	0.000	0.207	-9.36	-33.45	14.72	1.000	-11.30	-27.68	5.08	0.280	-1.94	-26.91	23.04	1.000	0.293
Time at 60-79 steps/min (min/day)	31.21	16.08	25	19.30	6.18	6	24.21	9.92	18	0.080	0.104	0.000	0.261	11.91	-3.05	26.87	0.162	7.00	-3.18	17.17	0.283	-4.91	-20.42	10.60	1.000	**0.043**
Time at 80-99 steps/min (min/day)	32.02	22.79	25	16.13	8.15	6	16.93	10.43	18	**0.016**	0.164	0.005	0.330	15.89	-4.26	36.05	0.169	15.09	1.38	28.79	**0.026**	-0.80	-21.70	20.10	1.000	**0.011**
Time at 100-119 steps/min (min/day)	20.47	11.02	25	22.30	28.01	6	13.93	10.57	18	0.241	0.060	0.000	0.199	-1.83	-17.40	13.73	1.000	6.54	-4.04	17.12	0.394	8.37	-7.77	24.51	0.612	0.275

Time at ≥120 steps/min (min/day)	8.56	9.12	25	1.73	2.47	6	2.34	3.46	18	**0.010**	0.183	0.013	0.350	6.83	-1.04	14.69	0.109	6.22	0.87	11.57	**0.018**	-0.61	-8.77	7.55	1.000	**0.017**
Time of moderate physical activity based on cadence (min/day)	24.59	13.80	25	23.18	27.80	6	14.91	10.74	18	0.117	0.089	0.000	0.241	1.40	-15.58	18.39	1.000	9.67	-1.87	21.22	0.129	8.27	-9.34	25.88	0.748	0.160
Time of vigorous physical activity based on cadence (min/day)	4.44	6.11	25	0.85	1.63	6	1.36	2.70	18	0.069	0.110	0.000	0.268	3.59	-1.76	8.95	0.307	3.09	-0.56	6.73	0.122	-0.51	-6.06	5.05	1.000	0.141
Time of moderate-vigorous physical activity based on cadence (min/day)	29.03	16.70	25	24.03	27.56	6	16.27	11.06	18	0.054	0.119	0.000	0.280	5.00	-13.68	23.67	1.000	12.76	0.06	25.46	**0.049**	7.76	-11.60	27.13	0.973	0.086
Sleep quality																										
Sleep duration (hours/night)	6.24	0.68	24	5.73	1.17	6	6.39	0.75	18	0.209	0.067	0.000	0.212	0.51	-0.37	1.39	0.47	-0.15	-0.75	0.46	1.00	-0.66	-1.57	0.25	0.24	0.220
WASO (hours/night)	1.41	0.57	24	1.87	1.59	6	1.09	0.47	18	0.072	0.110	0.000	0.270	-0.46	-1.29	0.37	0.52	0.32	-0.24	0.89	0.49	0.79	-0.07	1.64	0.08	0.069
Sustained Inactivity bouts during waking hours (hours/night)	1.62	0.80	24	1.68	0.32	6	1.66	0.75	18	0.973	0.001	0.000	0.003	-0.06	-0.90	0.78	1.00	-0.05	-0.62	0.53	1.00	0.01	-0.85	0.88	1.00	0.929
Sustained inactivity bouts during sleep period time (nº)	19.45	3.87	24	18.14	4.91	6	17.20	3.43	18	0.179	0.074	0.000	0.221	1.31	-3.05	5.68	1.00	2.25	-0.73	5.24	0.20	0.94	-3.57	5.45	1.00	0.107
Awakenings after sleep onset (nº)	18.45	3.87	24	17.14	4.91	6	16.20	3.43	18	0.179	0.074	0.000	0.221	1.31	-3.05	5.68	1.00	2.25	-0.73	5.24	0.20	0.94	-3.57	5.45	1.00	0.107
Sustained inactivity bouts during waking hours (nº)	7.71	3.42	24	7.38	2.61	6	7.88	3.82	18	0.955	0.002	0.000	0.025	0.32	-3.65	4.30	1.00	-0.17	-2.89	2.54	1.00	-0.50	-4.60	3.61	1.00	0.939
Sustained inactivity bouts during waking hours (hours/night)	0.83	0.59	24	0.96	0.34	6	0.90	0.50	18	0.828	0.008	0.000	0.081	-0.13	-0.74	0.47	1.00	-0.07	-0.48	0.34	1.00	0.07	-0.56	0.69	1.00	0.782
Duration of each sustained inactivity bout during waking hour (hours/night)	0.21	0.06	24	0.26	0.08	6	0.22	0.07	18	0.218	0.065	0.000	0.209	-0.05	-0.13	0.02	0.25	-0.01	-0.06	0.04	1.00	0.04	-0.03	0.12	0.54	0.246
Sleep regularity index	51.63	12.23	24	56.06	13.87	6	51.86	10.33	18	0.701	0.016	0.000	0.110	-4.43	-17.76	8.91	1.00	-0.23	-9.34	8.89	1.00	4.20	-9.58	17.97	1.00	0.764

Data represent mean and standard deviation, mean difference, and 95% confidence interval. Global P values and η^2 obtained from one-way analysis of variance (ANOVA) and between groups differences P values obtained after post-hoc Bonferroni correction. P values for the analysis of covariance (ANCOVA) adjusted for sex are also shown. Bold values mean statistically significant P values. *Abbreviations*: CI, confidence interval; LM, lean mass; MVPA, moderate-to-vigorous physical activity; RMR, resting metabolic rate; WASO, wake after sleep onset.

Table S6. Differences in subjective health and wellness between endurance, strength, and intermittent sports athletes.

	**Endurance vs. strength**				**Endurance vs. intermittent**				**Strength vs. intermittent**				**ANCOVA adjusted for sex**
	Mean difference	95% CI		P value	Mean difference	95% CI		P value	Mean difference	95% CI		P value	P value
ADAM-Q (Total score)	-0.01	-2.227	2.207	1.000	0.49	-1.252	2.233	1.000	0.50	-1.874	2.874	1.000	-
IPAQ	-1545.10	-3955.943	865.750	0.362	1013.00	-858.091	2884.092	0.566	2558.10	-6.669	5122.862	0.051	0.056
EAT-26 (Total score)	1.38	-5.060	7.825	1.000	-1.02	-5.915	3.876	1.000	-2.40	-9.272	4.469	1.000	0.688
SRS (Total score)	-0.02	-0.797	0.762	1.000	-0.22	-0.830	0.382	1.000	-0.21	-1.033	0.620	1.000	0.640
ASPS (Total score)	7.19	-1.691	16.075	0.153	1.07	-5.829	7.960	1.000	-6.13	-15.577	3.324	0.349	0.151
PSQI (Total score)	-1.22	-3.959	1.521	0.836	-1.42	-3.608	0.764	0.345	-0.20	-3.150	2.744	1.000	0.221
LEAF-Q (Total score)	2.57	-6.017	11.160	1.000	-5.15	-11.623	1.321	0.155	-7.72	-16.825	1.381	0.117	-
LEAF-Q (Injuries score)	-0.11	-4.075	3.861	1.000	-3.135	-6.125	-0.144	**0.038**	-3.03	-7.234	1.178	0.229	-
LEAF-Q (GI function score)	1.29	-2.631	5.202	1.000	-0.44	-3.388	2.515	1.000	-1.72	-5.873	2.429	0.889	-
LEAF-Q (Menstrual cycle score)	1.39	-2.273	5.059	1.000	-1.58	-4.342	1.183	0.463	-2.97	-6.858	0.914	0.182	-
NUKYA (Total score)	-0.62	-6.774	5.530	1.000	-1.94	-6.713	2.836	0.969	-1.32	-7.862	5.228	1.000	0.623
Overtraining (Total score)	-3.53	-11.911	4.858	0.917	-2.81	-9.042	3.424	0.818	0.72	-8.219	9.654	1.000	0.344
RESTQ-76 - General stress score	-0.07	-3.457	3.319	1.000	-1.55	-4.065	0.961	0.403	-1.48	-5.103	2.138	0.955	0.298
RESTQ-76 - Emotional stress score	-0.64	-3.578	2.290	1.000	-0.44	-2.615	1.737	1.000	0.20	-2.931	3.340	1.000	0.777
RESTQ-76 - Social stress score	-0.88	-3.814	2.055	1.000	-0.74	-2.921	1.432	1.000	0.13	-3.001	3.271	1.000	0.596
RESTQ-76 - Conflicts/pressure score	-0.24	-4.208	3.736	1.000	0.04	-2.904	2.988	1.000	0.28	-3.966	4.523	1.000	0.987
RESTQ-76 - Fatigue score	0.27	-3.521	4.059	1.000	-1.27	-4.086	1.537	0.810	-1.54	-5.594	2.507	1.000	0.467
RESTQ-76 - Lack of energy score	-0.31	-3.480	2.864	1.000	-0.70	-3.052	1.654	1.000	-0.39	-3.781	2.998	1.000	0.757
RESTQ-76 - Physical alterations score	0.85	-1.907	3.604	1.000	-0.49	-2.530	1.558	1.000	-1.33	-4.279	1.610	0.810	0.550
RESTQ-76 - Success score	-0.70	-5.190	3.790	1.000	-0.13	-3.461	3.200	1.000	0.57	-4.228	5.368	1.000	0.942
RESTQ-76 - Social recovery score	1.33	-3.158	5.819	1.000	-0.33	-3.663	2.995	1.000	-1.67	-6.462	3.131	1.000	0.705
RESTQ-76 - Physical recovery score	1.50	-2.664	5.669	1.000	-0.38	-3.466	2.715	1.000	-1.88	-6.331	2.575	0.913	0.571
RESTQ-76 - General well-being score	2.20	-2.577	6.982	0.786	0.49	-3.051	4.039	1.000	-1.71	-6.817	3.399	1.000	0.551
RESTQ-76 - Sleep quality score	-0.46	-4.289	3.360	1.000	-0.09	-2.923	2.751	1.000	0.38	-3.709	4.465	1.000	0.960
RESTQ-76 - Alterations of rest periods score	0.03	-2.749	2.816	1.000	0.72	-1.339	2.789	1.000	0.69	-2.282	3.665	1.000	0.685
RESTQ-76 - Emotional fatigue score	1.16	-2.162	4.490	1.000	-0.74	-3.207	1.726	1.000	-1.90	-5.458	1.650	0.579	0.418
RESTQ-76 Injuries score	-1.97	-5.769	1.831	0.623	-0.90	-3.718	1.919	1.000	1.07	-2.991	5.130	1.000	0.403
RESTQ-76 - Being in shape score	0.14	-4.512	4.795	1.000	0.16	-3.289	3.614	1.000	0.02	-4.951	4.995	1.000	0.988
RESTQ-76 - Personal fulfillment score	-1.62	-6.212	2.976	1.000	-0.46	-3.865	2.951	1.000	1.16	-3.749	6.070	1.000	0.670
RESTQ-76 - Self-efficacy score	-1.15	-6.058	3.755	1.000	0.64	-2.995	4.283	1.000	1.80	-3.447	7.039	1.000	0.715
RESTQ-76 - Self-regulation score	-0.49	-6.041	5.051	1.000	0.25	-3.865	4.362	1.000	0.74	-5.183	6.670	1.000	0.951

Data represent mean difference and 95% confidence interval. P values and from one-way analysis of variance (ANOVA) after post-hoc Bonferroni correction. P values for the analysis of covariance (ANCOVA) adjusted for sex are also shown. Bold values mean statistically significant P values. *Abbreviations*: ADAM-Q, Androgen Deficiency in the Aging Male Questionnaire; ASPS, Athlete's Subjective Performance Scale; CI, confidence interval; EAT-26, Eating Attitudes Test-26; GI, gastrointestinal; LEAF-Q, Low

Energy Availability in Females Questionnaire; NUKYA, Nutrition Knowledge for Young and Adult Athletes; PSQI, Pittsburgh Sleep Quality Index; RESTQ-76, Recovery-Stress Questionnaire for Athletes-76; SRS, Silhouette Rating Scale.
